# The Role of Excessive Anticoagulation and Missing Hyperinflammation in ECMO-Associated Bleeding

**DOI:** 10.3390/jcm11092314

**Published:** 2022-04-21

**Authors:** Sasa Rajsic, Robert Breitkopf, Ulvi Cenk Oezpeker, Zoran Bukumirić, Moritz Dobesberger, Benedikt Treml

**Affiliations:** 1 General and Surgical Intensive Care Unit, Department of Anaesthesiology and Critical Care Medicine, Medical University Innsbruck, 6020 Innsbruck, Austria; sasa.rajsic@i-med.ac.at (S.R.); mo.dobesberger@gmail.com (M.D.); benedikt.treml@tirol-kliniken.at (B.T.); 2 Transplant Surgical Intensive Care Unit, Department of Anaesthesiology and Critical Care Medicine, Medical University Innsbruck, 6020 Innsbruck, Austria; robert.breitkopf@tirol-kliniken.at; 3 Department of Cardiac Surgery, Medical University Innsbruck, 6020 Innsbruck, Austria; 4 Institute of Medical Statistics and Informatics, Faculty of Medicine, University of Belgrade, 11000 Belgrade, Serbia; zoran.bukumiric@med.bg.ac.rs

**Keywords:** anticoagulation, aPTT, bleeding, complications, extracorporeal life support, ECMO, inflammation, mortality

## Abstract

Extracorporeal membrane oxygenation (ECMO) is increasingly used in carefully selected patients with cardiac or respiratory failure. However, complications are common and can be associated with worse outcomes, while data on risk factors and outcomes are inconsistent and sparse. Therefore, we sought to investigate potential risk factors and predictors of haemorrhage and adverse events during ECMO and its influence on mortality. We retrospectively reviewed all patients on ECMO support admitted to intensive care units of a tertiary university centre in Austria. In a period of ten years, ECMO support was used in 613 patients, with 321 patients meeting the inclusion criteria of this study. Haemorrhage, occurring in more than one third of the included patients (123, 38%), represented the most common and serious ECMO complication, being associated with an increased one year mortality (51% vs. 35%, *p* = 0.005). The main risk factors for haemorrhage were severity of the disease (hazard ratio (HR) = 1.01, *p* = 0.047), a prolonged activated partial thromboplastin time (HR = 1.01, *p* = 0.007), and lower values of C-reactive protein (HR = 0.96, *p* = 0.005) and procalcitonin (HR = 0.99, *p* = 0.029). In summary, haemorrhage remained the main ECMO complication with increased mortality. Moreover, we reported a possible association of lower inflammation and bleeding during ECMO support for the first time. This generated a new hypothesis that warrants further research. Finally, we recommend stricter monitoring of anticoagulation especially in patients without hyperinflammation.

## 1. Introduction

The use of extracorporeal membrane oxygenation (ECMO) in patients with refractory cardiac or respiratory failure is increasing [1,2,3]. ECMO is a temporary mechanical extracorporeal support, aiming to support pulmonary or cardiac functions until recovery. It can be established as a venovenous (vv-ECMO) or venoarterial (va-ECMO) configuration. Additional possible uses of ECMO support include bridging to lung or heart transplant and rewarming of patients with severe hypothermia. Moreover, initiation of ECMO support through emergency medical service for out-of-hospital cardiac arrest has been popularised in the last decade [4,5]. According to the Extracorporeal Life Support Organization (ELSO) recommendations, ECMO support should be considered in cardiorespiratory failure with a mortality risk above 50%, and is indicated in a selected patient population with a mortality risk exceeding 80% [6].

Based on the data from 521 international ELSO registry centres, more than 154,000 ECMO runs were registered until the end of 2020, with 18 260 runs during the last year. The overall survival to hospital discharge or transfer was 54% [7].

While this type of extracorporeal support is lifesaving in selected patients, complications are still common and both haemorrhage and thrombosis are associated with reduced outcome [8,9], independent of the initial ECMO indication. Recently, a group of ECMO pioneers from the University of Michigan reported an overall bleeding incidence of 39% [10]. Intracranial haemorrhages or infarction represented a total of 8%. Therefore, improvement in prevention and early recognition of haemostatic complications including both haemorrhage and thrombosis remain pivotal to improve patient outcomes through better management of anticoagulation therapy.

In this study, we aimed to investigate potential risk factors and predictors of haemorrhage during ECMO support. Moreover, we provided a summary and comparison of the demographic and clinical characteristics of patients undergoing ECMO support, while focusing on adverse events and outcomes.

## 2. Materials and Methods

### 2.1. Patient Selection

We retrospectively reviewed the electronic medical records of all patients admitted to the trauma intensive care unit (ICU) and the general and surgical ICU of the Department of Anaesthesiology and Critical Care Medicine, Medical University Innsbruck, Austria. These tertiary ICUs treat surgical, post-trauma, and medical patients. The observation period included 10 years, from January 2010 to December 2019.

All patients undergoing ECMO were assessed for eligibility. Exclusion criteria were patients younger than 15 years, patients having their second ECMO initiation, and patients with incomplete data sets.

### 2.2. Data Collection

We obtained (1) socio-demographic data including age, sex, body weight, height, body mass index, and information regarding ICU ward; (2) data on disease severity as the simplified acute physiology score III (SAPS III) and sequential organ failure assessment (SOFA) score on ICU admission, mechanical cardiopulmonary resuscitation before or during ECMO implantation, underlying disease, indication and type of ECMO support, and duration of ECMO support; (3) detailed information on complications; (4) use of anticoagulation, transfusion of blood, and coagulation products; (5) coagulation status including platelet count (g/L), fibrinogen (modified Clauss method, mg/dL), factor XIII (%), rotational thromboelastometry (ROTEM^®^), prothrombin time (%), activated partial thromboplastin time (aPTT, seconds), international normalised ratio and antithrombin (%); (6) other laboratory parameters such as haemoglobin (g/L), haematocrit (l/L), erythrocytes (T/L), white blood cells (g/L), C-reactive protein (CRP, mg/dL), and procalcitonin (µg/L); and finally (7) cause and date of death with delivered data on ICU mortality, in-hospital mortality, and up to one year mortality.

Laboratory data were recorded starting 24 h before ECMO initiation and daily during the whole support period until a maximum of 14 days. The time frame was chosen based on the median duration of ECMO support (6 days) and percentage of patients (95%) having ECMO terminated within the 14 days. 

Two authors independently checked each electronic medical record and extracted the data in a predesigned case report form.

This retrospective study was approved by the Ethics Committee of the Medical University of Innsbruck, Austria (#1274/2019).

### 2.3. Anticoagulation Protocol

Management of elective patients under antithrombotic drugs was performed according to the national and international guidelines [11,12]. All patients receiving elective surgical procedures and on a dual platelet therapy continued using acetylsalicylic acid while P2Y12 inhibitors were paused before intervention. If Multiplate^®^ (Roche, Basel, Switzerland) intraoperatively showed presence of significant platelet blockade and satisfactory haemostasis could not be achieved, platelet concentrates were transfused during surgery.

Patients with new oral anticoagulants underwent elective procedures only with blood levels below threshold. In case of emergency procedures, the effects of these drugs were antagonised if possible. All patients with suspected acute coronary syndrome received loading with acetylsalicylic acid and 70 IU/kg unfractionated heparin (UFH) during prehospital treatment. P2Y12 inhibitors were administered—after consultation with the cardiologist on duty—either prehospital or immediately before percutaneous coronary intervention. Due to local protocols, none of our patients received ticagrelor.

Unfractionated heparin-coated ECMO circuits were used, and all patients received a loading dose of 50–100 IU/kg UFH before cannulation for ECMO, if they were not already on cardiopulmonary bypass. After ECMO initiation, anticoagulation was adapted according to the low-range activated clotting time (LR-ACT), aPTT, CT INTEM in the ROTEM^®^, and blood drug concentration or an anti-factor Xa assay activity. Patients with still inadequate haemostasis received substitution of coagulation factors in order to achieve adequate coagulation for ECMO support.

Anticoagulation of patients receiving ECMO support was conducted according to the local standard operating procedure protocol and the ELSO Anticoagulation Guideline [13]. Unfractionated heparin (initiated with 5–20 IU/kg/hour, with the targeted aPTT), was used as the first choice for anticoagulation. In the case of inadequate anticoagulation with UFH or suspected or proven heparin-induced thrombocytopenia type 2 (HIT 2), anticoagulation was changed to argatroban. In rare cases of increased coagulation (e.g., enhanced turn-over of concomitant renal replacement filters), epoprostenol (4 ng/kg/min) was used. In presence of severe coagulopathy, anticoagulation was paused. Continuous administration of UFH or argatroban was titrated to an aPTT value of 50–70 s and argatroban blood concentration of 0.3–0.5 µg/mL, respectively. Directly after anticoagulation initiation, monitoring was performed every 30 min until a stable aPTT or argatroban blood concentration was reached. After reaching a steady state, routine controls were performed every six hours and after every change of dosing until reaching stable conditions again. With every blood gas analysis, point-of-care ACT was measured, and aPTT was repeated if deteriorated.

### 2.4. Objectives and Outcomes

The primary endpoint of our work was identification of potential risk factors and predictors of haemorrhage during ECMO support. Secondary endpoints included the comparison (bleeding versus no bleeding event) of demographic and clinical characteristics as well as the incidence and type of adverse events during ECMO support. Finally, we evaluated the effect of haemorrhage on mortality and reported on subgroup analyses based on the type of ECMO, presence of surgical intervention, and major haemorrhage.

Reported outcomes comprised bleeding, thromboembolic events, sepsis, and mortality. Haemorrhagic complications were only observed during the period of ECMO support. Thereafter, bleeding events were considered as not being ECMO-related. Haemorrhagic events were defined as major or minor, according to the ELSO definition [13]. A major bleeding event was defined as clinically overt bleeding associated with a haemoglobin decrease of at least 2 g/dL over 24 h or administration of two or more red blood cell concentrate units over the same period [13]. Any pulmonary or retroperitoneal bleeding involving the central nervous system or requiring surgical intervention was also considered as major bleeding. Minor bleeding events were defined as any other noticeable bleeding [13]. Severe coagulopathy was defined as clinically significant bleeding with impaired clot formation and need for blood product substitution. We recorded only the date of the first bleeding event, in case if multiple bleeding events or sources appeared. Computed tomography was performed before ECMO support initiation (except in the case of ECMO implantation under resuscitation). Additional imaging for bleeding or thromboembolic events were performed if clinically indicated, e.g., in clinically suspected acute intracranial pathology. 

Information on thromboembolic events (date of identification, localisation, and type of thromboembolic complication) was gathered from the medical documentation and radiological reports during the ECMO support and within the two weeks after ECMO termination. The retrospective collection of data on the presence of thrombosis was only possible if radiological investigation was performed during the whole observation period. Thromboembolic events were confirmed using computed tomography or ultrasound. Thrombosis was stratified into central arterial and venous (heart, pulmonary artery, and aorta) or peripheral thrombus formation (all peripheral veins and arteries), embolization (i.e., ischaemic stroke), ECMO cannula or central vascular catheters, and mixed arterial and venous thrombosis. 

We recorded data on the date of death, and therefore calculated mortality in different periods. The information on patient death was collected from the hospital records.

### 2.5. Statistical Analyses

A statistician not involved in the study procedures or patient assessment performed the statistical analyses using SPSS (Version 22.0. Released 2013, Armonk, NY, USA: IBM Corp.) and R version 4.0.2 (free software for statistical computing and graphics—R Core Team 2020: a language and environment for statistical computing; R Foundation for Statistical Computing, Vienna, Austria). All statistical assessments were two-sided, and a significance level of 0.05 was used. Depending on the type of variables and the normality of the distribution, results were presented as frequency (percent), median (range), or mean with standard deviation. For parametric data, independent samples *t*-test was used, and for numeric data with non-normal distribution and ordinal data, Mann–Whitney U test was used. To test differences between nominal data (frequencies), chi-square test and Fisher’s exact test were used. In the univariate Cox proportional hazards model, we analysed the effect of each potential predictor of bleeding, and all significant covariates were assessed for the multivariate model. The significance level for the model was set to 0.1. The Kaplan-Meier method was used to estimate the time to bleeding event during ECMO support. To estimate the variability of observed laboratory values over the time (from ECMO support initiation until the event of interest), the coefficient of variation was calculated.

## 3. Results

During the observed period, 613 patients needed ECMO support. After screening all electronic medical charts, 415 patients met inclusion criteria, with 321 patients showing complete data sets. More than one third of included patients (123, 38%) experienced haemorrhage. 

The main indication for extracorporeal support was cardiogenic shock (223, 70%, Table 1 and Table 2). Before initiation of ECMO support, the median SAPS III was 67 (28–117). Cardiopulmonary resuscitation had to be performed in 19% (61) of patients. The median length of ICU stay was 18 (1–170) days.

Venoarterial ECMO configuration was used in three out of four patients (247, 77%), and vv-ECMO configuration was used in 74 (23%) patients (Table 2). The support was predominantly initiated on the day of ICU admission, with the vast majority during working days (255, 79%) and a median overall duration of 6 (1–36) days. Anticoagulation was realised with UFH (256, 80%), argatroban (30, 9%), and epoprostenol (1, 0.3%), and due to some type of coagulopathy, 29 (9%) patients were temporarily not anticoagulated. Finally, weaning from extracorporeal support was successful in 230 (72%) patients, and 197 (61%) were discharged from hospital.

### 3.1. Adverse Events during ECMO Support

**Figure 1:**
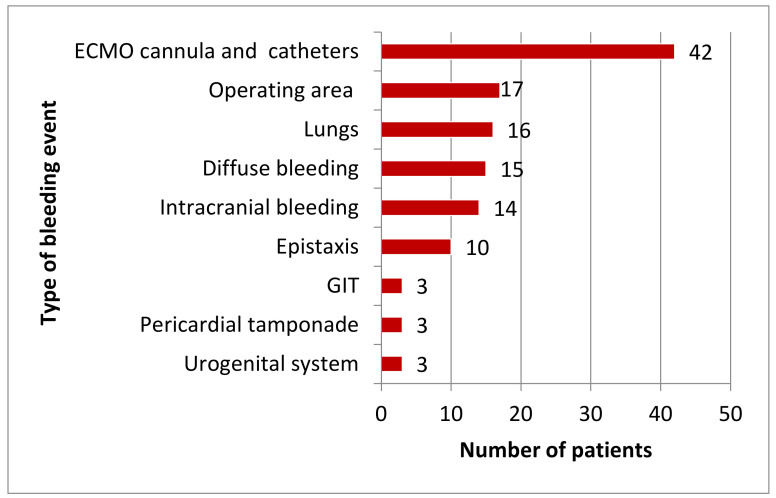
Type of bleeding event in patients receiving ECMO support (*n* = 123). ECMO: extracorporeal membrane oxygenation; GIT: gastrointestinal tract.

**Figure 2:**
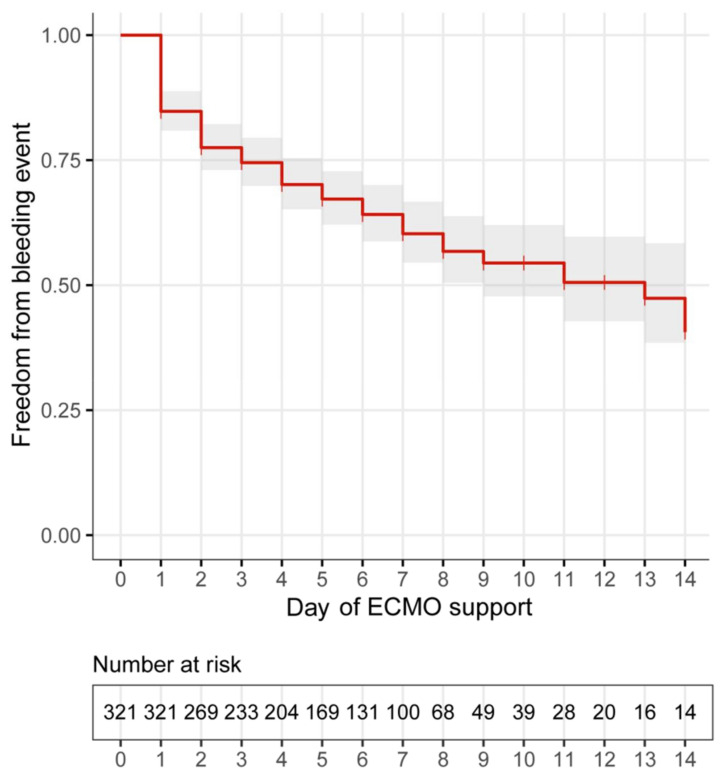
Kaplan-Meier curve: time from ECMO initiation to bleeding event (*n* = 321, median estimate 13 days, 95% CI 9.3–16.7). ECMO: extracorporeal membrane oxygenation.

The most common complication was haemorrhage (123, 38%), followed by thrombosis (74, 23%) and sepsis (67, 21%) (Table 2). Haemorrhage occurred at a median of 2 (1–14) days after ECMO support initiation, with similar incidences of major (60, 19%) and minor (62, 19%) events. Major bleedings were located most frequently in the surgical area (17, 14%), followed by the lung (16, 13%) and the cranium (14, 11%); minor bleedings occurred at the site of ECMO cannulation or central venous catheters (42, 34%) (Figure 1). The cumulative incidence of haemorrhage is depicted in Figure 2, with an estimated median time to a bleeding event of 13 days (95% CI 9.3–16.7).

### 3.2. Factors Associated with Bleeding Events

**Figure 3:**
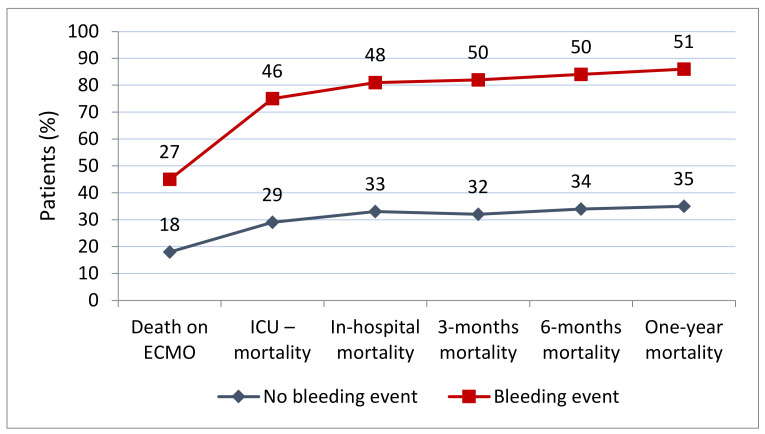
All-cause mortality in relation to time (*n* = 132): patients with bleeding events (red) and patients without bleeding events (blue). ECMO: extracorporeal membrane oxygenation; ICU: intensive care unit.

Patients with bleeding events had higher SAPS III (69 vs. 65, *p* = 0.023) and SOFA (13 vs. 12, *p* = 0.005) scores, lower inflammatory markers (C-reactive protein (5.5 vs. 7.7, *p* = 0.002) and procalcitonin (3.4 vs. 7.0, *p* < 0.001)), and a prolonged aPTT (58 vs. 56.5). ECMO support was needed longer (7 vs. 6 days), and fewer patients were successfully weaned from support or bridged to another support (Table 2). Furthermore, these patients received more blood products and coagulation factors (Table 3). Finally, patients with bleeding events had a higher mortality in all registered periods with cardiac failure being the main cause of death within 90 days (41, 33%), followed by multiple organ failure (38, 31%) and brain death (26, 21%) (Figure 3 and Table S1).

In the analysis of laboratory parameter fluctuations over the time on ECMO, higher variability of prothrombin time (HR = 1.02, *p* = 0.016) was associated with bleeding (Table S2).

Following univariate analyses (Table S3), SAPS III score, CRP, Fibrinogen, and aPTT were included in a multivariate analysis. The final model consisted of four variables, as the rest were excluded due to multicollinearity or large numbers of missing values. A higher SAPS III score (HR = 1.01; *p* = 0.047), lower values of CRP (HR = 0.96; *p* = 0.005), and a prolonged aPTT (HR = 1.01; *p* = 0.007) were associated with increased risk of bleeding (Table 4). In a further multivariate analysis, SOFA score and procalcitonin were included in the model (Table S4).

### 3.3. Subgroup Analyses

Comparison of critically ill patients regarding the ECMO configuration (va-ECMO or vv-ECMO) is shown in Tables S5–S7. In short, patients with respiratory failure were significantly younger (48 vs. 60, *p* < 0.001), less often resuscitated before support initiation (10% vs. 22%, *p* = 0.017), and had a longer ICU stay (21 vs. 17 days, *p* = 0.010). There was no difference in disease severity scores or ICU mortality. The univariate analysis of va-ECMO identified the same risk factors for bleeding as in the main analysis (Table S8), opposite to the vv-ECMO (Table S9). The multivariate model identified lower C-reactive protein (HR = 0.96; *p* = 0.020) and prolonged aPTT (HR = 1.01; *p* = 0.029) to be associated with an increased risk of bleeding (Table S10).

Another subgroup analysis compared patients with or without surgical procedures, except for ECMO cannulation. Patients without surgical procedures were younger (52 vs. 62, *p* < 0.001), more often resuscitated before ECMO initiation (27% vs. 12%, *p* = 0.001), had higher SAPS III scores (70 vs. 64, *p* < 0.001), and needed longer ECMO support (7 vs. 6 days, *p* = 0.003) (Table S11). Furthermore, these patients experienced more frequent bleeding events (44% vs. 33%, *p* = 0.046). The multivariate model identified lower C-reactive protein (HR = 0.96; *p* = 0.034) and prolonged aPTT (HR = 1.01; *p* = 0.004) to be associated with an increased risk of bleeding in patients without surgical interventions (Table S12).

Moreover, comparing patients with major bleeding to those without or with only minor bleeding events revealed higher SAPS III (69 vs. 66) and SOFA (13 vs. 12, *p* = 0.004) scores, ICU mortality (53% vs. 32%, *p* = 0.002), and more frequent coagulopathy and sepsis in patients with major bleeding (Table S13). In the Cox model, a higher SOFA score (HR = 1.08; *p* = 0.021) and presence of surgical intervention (HR = 1.88; *p* = 0.026) were associated with increased risk of bleeding (Table S14).

Finally, to analyse the influence of bleeding on the first ECMO day (to exclude direct postoperative bleeding), or ECMO duration less than two days on outcomes, a subgroup analysis was performed. However, this analysis did not show any significant differences as compared to the main analysis (data not shown).

## 4. Discussion

In this retrospective study from a Central European university centre, we reported on patient characteristics, risk factors for haemorrhage, and clinically important outcomes of critically ill patients needing ECMO support. Almost 40% of our patients experienced bleeding events during ECMO. These patients (1) were sicker, (2) spent more time on extracorporeal life support, and (3) had an increased mortality. Moreover, a prolonged aPTT and a weaker inflammatory response were associated with an increased bleeding rate. Finally, in our cohort, almost two thirds of the reasonably young patients survived utilising extracorporeal life support, with 61% of patients being discharged from the hospital.

Although ECMO support is lifesaving in many circumstances, complications are common and associated with the potential of permanent injury or even death. In our study, haemorrhage was the most frequent adverse event (38%), which is in line with a meta-analysis of 1763 patients reporting any kind of haemorrhage (40%) [14]. Major haemorrhage, as defined by the ELSO, occurred in 19%, and minor haemorrhage occurred in 20% of our patients. The cannulation and surgical area were the most common sites of bleeding, which is in line with current data [15,16,17]. From the 14 patients with an intracranial haemorrhage, 50% did not survive, which is comparable to findings from centres in the United Kingdom [8,18].

The overall survival to discharge was 61%, with a one year survival of 59% being in the higher share of the reported range (34–67%) [8,10,14,15,16,17,19]. Patients with haemorrhage had a higher mortality in all registered periods, corresponding to the literature [8,15,16] and being the highest in the case of intrapulmonary or intracranial bleeding.

### 4.1. Factors Associated with Haemorrhage

Several factors may be causative for haemorrhages during ECMO support, including vessel damage at the cannula or surgical sites, greater surgical complexity, or longer cardiopulmonary bypass times. Furthermore, coagulopathy due to reduced coagulation factors, thrombocytopenia, platelets dysfunction, acquired von Willebrand syndrome, or increased fibrinolysis may contribute to bleeding occurrence. Even fungal pneumonia or centrifugal ECMO pump have been shown to be associated with increased risk for haemorrhage [16,20,21,22,23,24,25]. Only some of these factors are modifiable, and whether their impact can be influenced by adaption of systemic anticoagulation or special therapeutic regime remains unclear [22].

In our study, we were able to identify a number of risk factors for haemorrhage during ECMO support. Higher SAPS III and SOFA scores, decreased clotting capability, and lower inflammation markers increased the risk of haemorrhage in the univariate analysis. Finally, the multivariate Cox regression model identified a higher SAPS III score (HR = 1.01, for every increase in one unit of measurement, hazard ratio increased 1%), prolonged aPTT (HR = 1.01), lower CRP (HR = 0.96), and procalcitonin (HR = 0.99) as predictors of bleeding in our retrospective study.

Prolonged aPTT has been inconsistently reported as a risk factor for bleeding during ECMO support [8,15,16,18], and our findings confirm earlier reports from Aubron et al. [16]. However, the ideal parameter for anticoagulation monitoring is still a matter of discussion, as different factors can influence anticoagulation monitoring in critically ill patients [13,26,27].

Interestingly, patients with a weaker inflammatory response had a higher risk of bleeding, which has not been reported in the literature. The association of inflammation and thrombosis (thromboinflammation) is well-established and discussed extensively in COVID-19 patients [28,29]. However, literature on the impact of inflammation on bleeding is still missing. Clearly, the activation of host defence results in activation of coagulation and a prothrombotic state [30,31,32,33,34]. The increased levels of both CRP and procalcitonin are allied with coagulation activation and consequent thrombosis [35,36].

Surgical trauma and exposure of blood to the artificial surface of the ECMO initiate and propagate the inflammatory response [37]. Inflammation further initiates clotting, decreases the activity of natural anticoagulant mechanisms, and impairs the fibrinolytic system in many different ways. Acute inflammation leads to an extensive elevation of the acute phase proteins and activation of diverse molecules. Within these processes, endotoxin, IL-1β, tumour necrosis factor-α (TNF-α), and neutrophil elastase reduce thrombomodulin on endothelial cell surfaces [38,39]. Furthermore, endothelial cell leucocyte adhesion molecules (P-selectin and E-selectin) are expressed on endothelial or platelet surfaces. Tissue factor is induced by endotoxin, TNF-α, or CD40 ligand on the cell surface of leucocytes, particularly monocytes, and it further binds factor VIIa, activates factor X, and forms complexes with factor Va to generate thrombin [40]. Additionally, inflammation decreases protein C levels, and inflammatory mediators increase the production of new and even more thrombogenic platelets [41,42]. This leads to the release of ultra-large von-Willebrand factor multimers and inhibits its cleavage by ADAMTS13 [43]. Increased CRP levels facilitate monocyte–endothelial cell interactions and promote plasminogen activator inhibitor-1 (PAI-1) and tissue factor formation with subsequent complement activation [44,45,46]; antithrombin is consumed and/or inactivated, and the concentration of vascular heparin-like molecules can be reduced by inflammatory cytokines and neutrophil activation products [47,48,49]. Finally, the role of the fibrinolytic system in ECMO patients is still not well-investigated. Recent data has shown that increased fibrinolytic activity during ECMO support is associated with an increased risk of bleeding [25,50]. In contrast, McVeen et al. reported on the normalisation of increased fibrinolytic enzymes within a few days after ECMO initiation [51]. In our study, we did not find an association of InTEM lysis index as measured by ROTEM^®^ and bleeding; however, further research in this area is warranted.

Given the above, the systemic anticoagulation of critically ill patients is complex and difficult, especially if done by UFH, a mixture of heterogeneous glycosaminoglycans of different molecular weights. Heparin’s polyanionic nature limits its interaction specificity with antithrombin, forming the heparin–antithrombin complex, which is a direct inhibitor of thrombin and factor Xa [52]. Hence, the steady-state plasma concentration is in no direct linear relation to dosage under continuous infusion [53]. Hyperinflammation can lead to a limitation of the anticoagulant effect of UFH by increasing the heparin-binding acute phase proteins, including factor VIII and fibrinogen, or decreasing the antithrombin levels [47,48,49]. Traditionally, anticoagulation monitoring is based on aPTT assays, despite the known heterogeneous results and the risk of interference in critically ill patients [26,48,54].

We hypothesised that in cases of a weak inflammatory response, prothrombotic activation by contact with artificial surfaces in the extracorporeal circuit would be less pronounced. This would lead to an increased susceptibility of bleeding under standard anticoagulation protocols which do not consider inflammatory response but rely only on aPTT, anti-factor Xa, or the blood concentration of the anticoagulant used. Lastly, this was limited by the fact that the ideal parameter for anticoagulation monitoring is still missing.

Finally, the extent of the inflammatory response to the ECMO circuit showed a high interpatient variability without a physiological rationale [37]. Hyperinflammation has a major impact on coagulation and heparinisation, and our hypothesis was that the unintended excessive anticoagulation in case of “non-hyperinflammation” would lead to the outcome-relevant bleeding.

### 4.2. Further Directions

Future research on risk factors for bleeding in regard to surgical techniques, ECMO systems, anticoagulation possibilities, and patient factors is warranted. The ongoing technological development of ECMO circuits, through different coating materials or modifications of extracorporeal circulation systems, resulted in reduced levels of thrombin [37]. Research on ECMO-induced inflammation and its association with clinical outcomes may lead to novel anti-inflammatory therapies with a more stable anticoagulation [55,56,57]. The ideal anticoagulant, with reduced or even eliminated risk of thrombosis and absent haemorrhage risk, is still not available. Emerging preclinical data suggest that antibodies targeting factor XI and XII may have improved safety and efficacy, but data in humans are still missing [55,56,57]. Whilst it is certainly difficult to isolate the relative contribution of specific patient factors to bleeding during ECMO support, failure to do so can result in a missed opportunity for intervention. Patient factors were extensively investigated, but due to the diversity in reporting and missing of guidelines on minimal reporting criteria for ECMO complications, a comparison of studies was challenging.

Further studies should investigate whether the consideration of the inflammatory response can farther personalise anticoagulation and reduce the incidence of bleeding events during ECMO. This is even more interesting given the rise of ECMOs in coronavirus disease 2019 (COVID-19) patients with a tendency towards a hypercoagulatory state. The ongoing prospective trials (ECLS-SHOCK, NCT03637205; EUROSHOCK, NCT03813134; ECMO-CS, NCT02301819; and ANCHOR, NCT04184635) aim to provide prospective information on the benefits of extracorporeal circulation in cardiac shock and respiratory failure. However, the gap between the association of inflammation and bleeding is still to be closed. We therefore recommend cautious monitoring (as per existing protocols) and adaptation of anticoagulation in patients with a weak inflammatory response until further evidence is available.

### 4.3. Limitations

This study was limited in several aspects. We reported on a mixed population of critically ill, all with a common attribute of a very high mortality risk, where ECMO support was initiated as the last resort. Due to the retrospective nature, selection bias could not be excluded. Furthermore, our analyses may be confounded by undisclosed or undiscovered factors such as the plasma levels of laboratory parameters being diluted by other drugs or other similar factors. However, as all of our patients experienced a distinct decrease in the majority of parameters, the chance for a significant confounding factor in only one of the groups should be rather small. We used the widely accepted ELSO bleeding definition to classify bleeding [13], but some complications may have been overlooked or missed. Given the liberal approach to diagnostic imaging and post-mortem examinations, this chance seems rather small. Although our study involved a quite large cohort of patients as compared to the literature, larger samples and prospective studies are needed to further elucidate the interaction of inflammation and haemostasis during ECMO support.

## 5. Conclusions

To the best of our knowledge, this was the largest European study investigating risk factors for bleeding events during ECMO support. Haemorrhage remains the most frequent and serious ECMO complication with an increased mortality. We confirmed previous findings of a prolonged aPTT and disease severity as risk factors for haemorrhage. For the first time, we reported on the association of inflammation and bleeding during ECMO support, generating a new hypothesis and warranting further research. Finally, based on our findings, we recommend stricter monitoring of anticoagulation especially in patients without hyperinflammation, until the further evidence is available.

## Figures and Tables

**Table 1:** Extracorporeal membrane oxygenation: patient demographic and clinical characteristics (*n* = 321).

Patient Characteristics		All Patients (*n* = 321)	No Bleeding Event (*n* = 198)	Bleeding Event (*n* = 123)	*p*-Value	Missing Data (*n*/Total)
Age (years)		57.5 ± 16.1	57.9 ± 16.1	56.7 ± 16.1	0.515	0/321
	<30	25 (7.8)	16 (8.1)	9 (7.3)	0.256	0/321
	31–45	39 (12.1)	22 (11.1)	17 (13.8)		
	46–60	99 (30.8)	59 (29.8)	40 (32.5)		
	61–75	124 (38.6)	75 (37.9)	49 (39.8)		
	>76	34 (10.6)	26 (13.1)	8 (6.5)		
Male sex		229 (71.3)	141 (71.2)	88 (71.5)	0.949	0/321
Height (cm)		173 ± 10.0	173 ± 8.7	171 ± 11.8	0.295	10/321
Weight (kg)		81.6 ± 17.9	82.0 ± 17.4	80.8 ± 18.7	0.566	10/321
Body mass index (kg/m^2^)		27.3 ± 5.3	27.3 ± 5.3	27.2 ± 5.3	0.845	10/321
SAPS III score (points)		67 (28–117)	65 (28–112)	69 (28–117)	0.023	1/321
SAPS III-score-predicted mortality (%)		50 (1–96)	46 (1–95)	54 (1–96)	0.023	1/321
SOFA score (points)		12 (2–21)	12 (2–21)	13 (4–21)	0.005	0/321
	SOFA respiratory	2 (0–4)	2 (0–4)	3 (0–4)	0.016	
	SOFA coagulation	1 (0–4)	1 (0–4)	1 (0–3)	0.270	
	SOFA liver	0 (0–4)	0 (0–4)	1 (0–4)	0.045	
	SOFA cardiovascular	4 (0–4)	4 (0–4)	4 (0–4)	0.076	
	SOFA neurology	4 (0–4)	4 (0–4)	4 (0–4)	0.092	
	SOFA renal	1 (0–4)	1 (0–4)	1 (0–4)	0.070	
CPR before ECMO initiation		61 (19.0)	35 (17.7)	26 (21.1)	0.442	0/321
Length of ICU stay (days)		18 (1–170)	18 (2–170)	17 (1–98)	0.576	0/321
ICU admission reason						0/321
	Respiratory failure	79 (24.6)	49 (24.7)	30 (24.4)	0.999	
	Cardiac nonsurgical	166 (51.7)	102 (51.5)	64 (52.0)		
	Cardiac surgery	61 (19.0)	37 (18.7)	24(19.5)		
	Trauma	3 (0.9)	2 (1.0)	1 (0.8)		
	Hypothermi	12 (4.0)	8 (4.0)	4 (3.3)		
ICU department						0/321
	ICU 1	178 (55.5)	112 (56.6)	66 (53.7)	0.610	
	ICU 2	143 (44.5)	86 (43.4)	57 (46.3)		
Mortality-related outcomes						0/321
	Time from admission to death within 90 days (days)	10 (1–88)	9.5 (2–79)	11.5 (1–88)	0.457	
	ICU mortality	115 (35.8)	58 (29.3)	57 (46.3)	0.002	

Data presented as mean ± standard deviation, median (minimum—maximum range), or number of patients (%). Abbreviations: SAPS III: simplified acute physiology score III; SOFA: sequential organ failure assessment score; ICU: intensive care unit; ECMO: extracorporeal membrane oxygenation; CPR: cardiopulmonary resuscitation; ICU 1: general and surgical ICU; and ICU 2: traumatology ICU.

**Table 2:** ECMO related characteristics and complications (*n* = 321).

Clinical Characteristics		All Patients (*n* = 321)	No Bleeding Event (*n* = 198)	Bleeding Event (*n* = 123)	*p*-Value	Missing Data (*n*/Total)
ECMO indications						0/321
	Cardiogenic shock	223 (69.5)	136 (68.7)	87 (70.7)	0.928	
	Respiratory failure	87 (27.1)	55 (27.8)	32 (26.0)		
	Hypothermia	11 (3.4)	7 (3.5)	4 (3.3)		
Type of ECMO support						0/321
	Venoarterial	247 (76.9)	154 (77.8)	93 (75.6)	0.654	
	Venovenous	74 (23.1)	44 (22.2)	30 (24.4)		
ECMO related clinical course						0/321
	ECMO support duration (days)	6 (1–36), mean 7.3	6 (1–30), mean 6.8	7 (1–36), mean 8.2	0.053	
	ECMO support duration < 7 days	209 (65.1)	141 (71.2)	68 (55.3)	0.004	
	Time from admission to ECMO initiation (days)	0 (0–36)	0 (0–17)	0 (0–36)	0.773	
Day of ECMO initiation						0/321
	Weekday	255 (79.4)	153 (77.3)	102 (82.9)	0.223	
	Weekend	66 (20.6)	45 (22.7)	21 (17.1)		
Anticoagulation during ECMO support						1/321
	None	29 (9.1)	15 (7.6)	14 (11.4)	0.465	
	UFH	256 (80.0)	162 (82.2)	94 (76.4)		
	Argatroban	30 (9.4)	17 (8.6)	13(10.6)		
	Epoprostenol	1 (0.3)	0 (0)	1 (0.8)		
	Argatroban and epoprostenol	4 (1.3)	3 (1.5)	1 (0.8)		
Complications						
	Major haemorrhage	60 (18.7)	-	60 (48.8)		0/123
	Minor haemorrhage	62 (19.3)	-	62 (50.4)		0/123
	Day of haemorrhage	-	-	2 (1–14), mean 3.2		0/123
	Haemorrhage at first ECMO day	52 (16.2)	-	52 (42.3)		0/123
	Haemorrhage within first three ECMO support days	117 (36.4)	-	117 (95.1)		0/123
	Coagulopathy	41 (12.8)	19 (9.6)	22 (17.9)	0.031	27/321
	Thrombosis	74 (23.1)	47 (23.7)	27 (22.0)	0.712	0/321
	Sepsis	67 (20.9)	36 (18.2)	31 (25.2)	0.132	0/321
Reason for termination of ECMO support						0/321
	Improvement (weaned)	230 (71.7)	148 (74.7)	82 (66.7)	0.002	
	Bridge to other assistance (heart transplant or ventricular assist device)	17 (5.3)	13 (6.6)	4 (3.3)		
	Haemorrhage	7 (2.2)	-	7 (5.7)		
	Death	67 (20.9)	37 (18.7)	30 (24.4)		

Data presented as median (minimum—maximum range) or number of patients (%). For clarity, mean was added if median was 0 and *p* value < 0.05. Abbreviations: ECMO: extracorporeal membrane oxygenation. UFH: unfractionated heparin.

**Table 3:** Laboratory parameters within 24 h prior to bleeding event and blood products substitution during ECMO support (*n* = 321).

	All Patients (*n* = 321)	No Bleeding Event (*n* = 198)	Bleeding Event (*n* = 123)	*p*-Value	Missing Data (*n*/Total)
Haemoglobin (g/dL)	92.6 ± 13.3	91.0 ± 7.7	94.4 ± 17.7	0.054	13/321
Red blood cells (T/L)	3.2 ± 0.5	3.2 ± 0.4	3.2 ± 0.6	0.952	13/321
Haematocrit (%)	0.3 ± 0.1	0.3 ± 0.1	0.3 ± 0.1	0.947	13/321
Leucocytes (g/L)	10.2 (1.3–71.7)	10.2 (1.5–71.7)	10.3 (1.3–29.6)	0.899	13/321
C-reactive protein (mg/L)	7.0 (0.1–35.5)	7.7 (0.1–35.5)	5.5 (0.1–35.1)	0.002	15/321
Procalcitonin (µg/L)	5.0 (0.1–1272.4)	7.0 (0.1–1272.4)	3.4 (0.1–118.9)	<0.001	30/321
Platelets (g/L)	87.0 (14–309)	89.0 (18–309)	84.5 (14–276)	0.373	13/321
International normalised ratio	1.5 (0.8–6)	1.5 (1.0–6)	1.4 (0.8–6.0)	0.422	11/321
Activated partial thromboplastin time (s)	58.0 (28–201)	56.5 (28–201)	58.0 (32–201)	0.255	21/321
Prothrombin time (%)	51.0 (9–104)	49.0 (9–104)	54.0 (9–101)	0.625	10/321
Fibrinogen (mg/dL)	256.5 (39–1053)	258.0 (44–1053)	251.0 (39–941)	0.226	15/321
Antithrombin (%)	45.0 (19–118)	43.0 (19–92)	51.0 (19–118)	0.018	14/321
Factor XIII (%)	59.0 (13–131)	59.0 (13–131)	60.0 (29–129)	0.500	188/321
InTEM clotting time (s)	211 (54–671)	204.5 (151–413)	214 (54–671)	0.350	191/321
InTEM maximal clot firmness (mm)	49 (5–71)	48 (25–71)	51 (5–68)	0.982	216/321
FibTEM maximal clot firmness (mm)	14 (2–31)	13.5 (3–31)	14 (2–30)	0.432	217/321
Substitution of blood products during ECMO support					
Packed red blood cells (units)	5 (0–60)	4 (0–36)	8 (0–60)	<0.001	15/321
Fresh-frozen plasma (units)	0 (0–92), mean 2.7	0 (0–27), mean 1.8	0 (0–92), mean 4.3	0.011	15/321
Platelets (units)	1 (0–30), mean 2.0	0 (0–15), mean 1.3	1 (0–30), mean 3.2	<0.001	15/321
Fibrinogen (g)	0 (0–26), mean 2.8	0 (0–22), mean 1.8	1 (0–26), mean 4.4	<0.001	15/321
Antithrombin (IU)	0 (0–32,000), mean 614.5	0 (0–16,500), mean 505.7	0 (0–32,000), mean 790.5	0.164	15/321
Prothrombin complex concentrate (IU)	0 (0–7200), mean 386.0	0 (0–6000), mean 285.4	0 (0–7200), mean 548.0	0.020	15/321
Factor XIII concentrate (IU)	0 (0–10,000), mean 570.0	0 (0–6250), mean 246.2	0 (0–10,000), mean 1096.3	<0.001	15/321
Desmopressin (µg)	0 (0–30), mean 0.6	0 (0–30), mean 0.3	0 (0–30), mean 1.1	0.070	18/321
Von Willebrand factor (IU)	0 (0–5000), mean 100.3	0 (0–2000), mean 10.1	0 (0–5000), mean 245.5	<0.001	27/321

Data presented as mean ± standard deviation, median (minimum—maximum range), or number of patients (%). For clarity, mean was added if median was 0 and *p* value < 0.05. Abbreviations: ECMO: extracorporeal membrane oxygenation; IU: international units.

**Table 4:** Identification of risk factors for bleeding: Cox multivariate analysis (*n* = 321).

Variable	B-Coefficient	*p*-Value	HR	95% Confidence Interval	
				Lower	Upper
SAPS III score	0.012	0.047	1.01	1.00	1.03
C-reactive protein (mg/L)	−0.046	0.005	0.96	0.93	1.00
aPTT (s)	0.006	0.007	1.01	1.00	1.01
Fibrinogen (mg/dL)	0.001	0.176	1.00	1.00	1.00

Abbreviations: SAPS III: simplified acute physiology score III; aPTT: activated partial thromboplastin time. (Cases with missing data: 21/321).

## Data Availability

The datasets used and analysed during the current study can be made available from the corresponding author on reasonable request.

## Supplementary Materials

The following supporting information can be downloaded at: https://www.mdpi.com/article/10.3390/jcm11092314/s1. 1. Main analysis: Table S1. ECMO support one year mortality-related outcomes (*n* = 321), Table S2. Variability of laboratory values during the ECMO support period (*n* = 321), Table S3. Risk factors for bleeding during ECMO support: univariate analysis (*n* = 321), Table S4. Risk factors for bleeding, model including SOFA score and procalcitonin: Cox multivariate analysis (*n* = 321); 2. Subgroup analysis: Comparison of venoarterial and venovenous ECMO configuration: Table S5. Extracorporeal membrane oxygenation: patient demographic and clinical characteristics, comparison of venoarterial and venovenous configuration (*n* = 321), Table S6. ECMO related characteristics and complications, comparison of venoarterial and venovenous configuration (*n* = 321), Table S7. Laboratory parameters within 24 h prior to bleeding event and blood products substitution in regard to ECMO configuration (*n* = 321), Table S8. Risk factors for bleeding: venoarterial ECMO configuration—univariate analysis, Table S9. Risk factors for bleeding: venovenous ECMO configuration—univariate analysis, Table S10. Risk factors for bleeding in regard to EMCO configuration: Cox multivariate analysis; 3. Subgroup analysis: Comparison of ECMO patients with and without surgical intervention: Table S11. ECMO related characteristics and complications, comparison of patients with and without surgical intervention (*n* = 321), Table S12. Risk factors for bleeding in regard to surgical intervention presence—Cox multivariate analysis (based on the univariate analysis, data not shown; *n* = 321); 4. Subgroup analysis: comparison of ECMO patients with major bleeding and minor or no bleeding event: Table S13. Major bleeding versus no or minor bleeding event—patient demographic and clinical characteristics (*n* = 321), Table S14. Risk factors for bleeding—major bleeding versus no or minor bleeding event; Cox multivariate analysis (based on the univariate analysis, data not shown; *n* = 321).

## References

[1] Biancari F., Mariscalco G., Dalén M., Settembre N., Welp H., Perrotti A., Wiebe K., Leo E., Loforte A., Chocron S. (2021). Six-Month Survival After Extracorporeal Membrane Oxygenation for Severe COVID-19. J. Cardiothorac. Vasc. Anesth..

[2] Lawler P.R., Silver D.A., Scirica B.M., Couper G.S., Weinhouse G.L., Camp P.C. (2015). Extracorporeal membrane oxygenation in adults with cardiogenic shock. Circulation.

[3] Munshi L., Walkey A., Goligher E., Pham T., Uleryk E.M., Fan E. (2019). Venovenous extracorporeal membrane oxygenation for acute respiratory distress syndrome: A systematic review and meta-analysis. Lancet Respir. Med..

[4] Inoue A., Hifumi T., Sakamoto T., Kuroda Y. (2020). Extracorporeal Cardiopulmonary Resuscitation for Out-of-Hospital Cardiac Arrest in Adult Patients. J. Am. Heart Assoc..

[5] Bougouin W., Dumas F., Lamhaut L., Marijon E., Carli P., Combes A., Pirracchio R., Aissaoui N., Karam N., Deye N. (2020). Extracorporeal cardiopulmonary resuscitation in out-of-hospital cardiac arrest: A registry study. Eur. Heart J..

[6] Extracorporeal Life Support Organization (ELSO) Guidelines for Cardiopulmonary Extracorporeal Life Support. https://www.elso.org/Portals/0/ELSO%20Guidelines%20For%20Adult%20Respiratory%20Failure%201_4.pdf.

[7] Extracorporeal Life Support Organization (ELSO) Registry Report on Extracorporeal Life Support, International Summary. https://www.elso.org/Registry/Statistics/InternationalSummary.aspx.

[8] Stokes J.W., Gannon W.D., Sherrill W.H., Armistead L.B., Bacchetta M., Rice T.W., Semler M.W., Casey J.D. (2020). Bleeding, Thromboembolism, and Clinical Outcomes in Venovenous Extracorporeal Membrane Oxygenation. Crit. Care Explor..

[9] Winkler A.M. (2017). Managing the Precarious Hemostatic Balance during Extracorporeal Life Support: Implications for Coagulation Laboratories. Semin. Thromb. Hemost..

[10] Gray B.W., Haft J.W., Hirsch J.C., Annich G.M., Hirschl R.B., Bartlett R.H. (2015). Extracorporeal life support: Experience with 2,000 patients. ASAIO J..

[11] Capodanno D., Angiolillo D.J. (2013). Management of Antiplatelet Therapy in Patients with Coronary Artery Disease Requiring Cardiac and Noncardiac Surgery. Circulation.

[12] Schlimp C.B.Z., Schöchl H., Alber H. (2018). Empfehlung der Arbeitsgruppe Perioperative Gerinnung der ÖGARI zum Thema: Perioperatives Management von PatientInnen mit Koronarstents unter Dualer Plättchenhemmung bei Nicht-Kardiochirurgischen Eingriffen.

[13] Lequier L.A.G., Al-Ibrahim O., Bembea M., Brodie D., Brogan T., Buckvold S., Chicoine L., Conrad S., Cooper D., Dalton H. (2014). ELSO Anticoagulation Guideline.

[14] Zangrillo A., Landoni G., Biondi-Zoccai G., Greco M., Greco T., Frati G., Patroniti N., Antonelli M., Pesenti A., Pappalardo F. (2013). A meta-analysis of complications and mortality of extracorporeal membrane oxygenation. Crit. Care Resusc..

[15] Oude Lansink-Hartgring A., de Vries A.J., Droogh J.M., van den Bergh W.M. (2019). Hemorrhagic complications during extracorporeal membrane oxygenation—The role of anticoagulation and platelets. J. Crit. Care.

[16] Aubron C., DePuydt J., Belon F., Bailey M., Schmidt M., Sheldrake J., Murphy D., Scheinkestel C., Cooper D.J., Capellier G. (2016). Predictive factors of bleeding events in adults undergoing extracorporeal membrane oxygenation. Ann. Intensive Care.

[17] Thiagarajan R.R., Barbaro R.P., Rycus P.T., McMullan D.M., Conrad S.A., Fortenberry J.D., Paden M.L. (2017). Extracorporeal Life Support Organization Registry International Report 2016. ASAIO J..

[18] Arachchillage D.J., Rajakaruna I., Scott I., Gaspar M., Odho Z., Banya W., Vlachou A., Isgro G., Cagova L., Wade J. (2021). Impact of major bleeding and thrombosis on 180-day survival in patients with severe COVID-19 supported with veno-venous extracorporeal membrane oxygenation in the United Kingdom: A multicentre observational study. Br. J. Haematol..

[19] Vakil D., Soto C., D’Costa Z., Volk L., Kandasamy S., Iyer D., Ikegami H., Russo M.J., Lee L.Y., Lemaire A. (2021). Short-term and intermediate outcomes of cardiogenic shock and cardiac arrest patients supported by venoarterial extracorporeal membrane oxygenation. J. Cardiothorac. Surg..

[20] Lotz C., Streiber N., Roewer N., Lepper P.M., Muellenbach R.M., Kredel M. (2017). Therapeutic Interventions and Risk Factors of Bleeding During Extracorporeal Membrane Oxygenation. ASAIO J..

[21] Halaweish I., Cole A., Cooley E., Lynch W.R., Haft J.W. (2015). Roller and Centrifugal Pumps: A Retrospective Comparison of Bleeding Complications in Extracorporeal Membrane Oxygenation. ASAIO J..

[22] Werho D.K., Pasquali S.K., Yu S., Donohue J., Annich G.M., Thiagarajan R.R., Hirsch-Romano J.C., Gaies M.G. (2015). Hemorrhagic complications in pediatric cardiac patients on extracorporeal membrane oxygenation: An analysis of the Extracorporeal Life Support Organization Registry. Pediatric Crit. Care Med..

[23] Tauber H., Ott H., Streif W., Weigel G., Loacker L., Fritz J., Heinz A., Velik-Salchner C. (2015). Extracorporeal membrane oxygenation induces short-term loss of high-molecular-weight von Willebrand factor multimers. Anesth. Analg..

[24] Kalbhenn J., Schmidt R., Nakamura L., Schelling J., Rosenfelder S., Zieger B. (2015). Early diagnosis of acquired von Willebrand Syndrome (AVWS) is elementary for clinical practice in patients treated with ECMO therapy. J. Atheroscler. Thromb..

[25] Doyle A.J., Hunt B.J. (2018). Current Understanding of How Extracorporeal Membrane Oxygenators Activate Haemostasis and Other Blood Components. Front. Med..

[26] Bachler M., Niederwanger C., Hell T., Höfer J., Gerstmeyr D., Schenk B., Treml B., Fries D. (2019). Influence of factor XII deficiency on activated partial thromboplastin time (aPTT) in critically ill patients. J. Thromb. Thrombolysis.

[27] Rajsic S., Breitkopf R., Bachler M., Treml B. (2021). Diagnostic Modalities in Critical Care: Point-of-Care Approach. Diagnostics.

[28] Ranucci M., Ballotta A., Di Dedda U., Baryshnikova E., Dei Poli M., Resta M., Falco M., Albano G., Menicanti L. (2020). The procoagulant pattern of patients with COVID-19 acute respiratory distress syndrome. J. Thromb. Haemost..

[29] Lim M.S., McRae S. (2021). COVID-19 and immunothrombosis: Pathophysiology and therapeutic implications. Crit. Rev. Oncol. Hematol..

[30] Connors J.M., Levy J.H. (2020). COVID-19 and its implications for thrombosis and anticoagulation. Blood.

[31] Engelmann B., Massberg S. (2013). Thrombosis as an intravascular effector of innate immunity. Nat. Rev. Immunol..

[32] Delabranche X., Helms J., Meziani F. (2017). Immunohaemostasis: A new view on haemostasis during sepsis. Ann. Intensive Care.

[33] Jackson S.P., Darbousset R., Schoenwaelder S.M. (2019). Thromboinflammation: Challenges of therapeutically targeting coagulation and other host defense mechanisms. Blood.

[34] Stark K., Massberg S. (2021). Interplay between inflammation and thrombosis in cardiovascular pathology. Nat. Rev. Cardiol..

[35] Gro G., Trond I., Ynse Ieuwe Gerardus Vladimir T., Jan B., Sigrid Kufaas B., John-Bjarne H. (2018). C-reactive protein and risk of venous thromboembolism: Results from a population-based case-crossover study. Haematologica.

[36] Asoğlu R., Tibilli H., Afşin A., Türkmen S., Barman H.A., Asoğlu E. (2020). Procalcitonin is a predictor of disseminated intravascular coagulation in patients with fatal COVID-19. Eur. Rev. Med. Pharmacol. Sci..

[37] Millar J.E., Fanning J.P., McDonald C.I., McAuley D.F., Fraser J.F. (2016). The inflammatory response to extracorporeal membrane oxygenation (ECMO): A review of the pathophysiology. Crit. Care.

[38] Moore K.L., Esmon C.T., Esmon N.L. (1989). Tumor necrosis factor leads to the internalization and degradation of thrombomodulin from the surface of bovine aortic endothelial cells in culture. Blood.

[39] Conway E.M. (2012). Thrombomodulin and its role in inflammation. Semin. Immunopathol..

[40] Lindmark E., Tenno T., Siegbahn A. (2000). Role of platelet P-selectin and CD40 ligand in the induction of monocytic tissue factor expression. Arterioscler. Thromb. Vasc. Biol..

[41] Van de Wouwer M., Collen D., Conway E.M. (2004). Thrombomodulin-protein C-EPCR system: Integrated to regulate coagulation and inflammation. Arterioscler. Thromb. Vasc. Biol..

[42] Iba T., Levy J.H. (2018). Inflammation and thrombosis: Roles of neutrophils, platelets and endothelial cells and their interactions in thrombus formation during sepsis. J. Thromb. Haemost..

[43] Bernardo A., Ball C., Nolasco L., Moake J.F., Dong J.F. (2004). Effects of inflammatory cytokines on the release and cleavage of the endothelial cell-derived ultralarge von Willebrand factor multimers under flow. Blood.

[44] Han K.H., Hong K.H., Park J.H., Ko J., Kang D.H., Choi K.J., Hong M.K., Park S.W., Park S.J. (2004). C-reactive protein promotes monocyte chemoattractant protein-1--mediated chemotaxis through upregulating CC chemokine receptor 2 expression in human monocytes. Circulation.

[45] Devaraj S., Xu D.Y., Jialal I. (2003). C-reactive protein increases plasminogen activator inhibitor-1 expression and activity in human aortic endothelial cells: Implications for the metabolic syndrome and atherothrombosis. Circulation.

[46] Wolbink G.J., Bossink A.W., Groeneveld A.B., de Groot M.C., Thijs L.G., Hack C.E. (1998). Complement activation in patients with sepsis is in part mediated by C-reactive protein. J. Infect. Dis..

[47] Esmon C.T. (2005). The interactions between inflammation and coagulation. Br. J. Haematol..

[48] Levy J.H., Connors J.M. (2021). Heparin Resistance—Clinical Perspectives and Management Strategies. New Engl. J. Med..

[49] Mulloy B., Hogwood J., Gray E., Lever R., Page C.P. (2016). Pharmacology of Heparin and Related Drugs. Pharmacol. Rev..

[50] Malhotra Kapoor P., Karanjkar A., Bhardwaj V. (2018). Evaluation of coagulopathy on veno-arterial ECMO (VA) extracorporeal membrane oxygenation using platelet aggregometry and standard tests: A narrative review. Egypt. J. Crit. Care Med..

[51] McVeen R.V., Lorch V., Carroll R.C., Goldberg L., Keszler M., Podlasek S., Stewart D.L. (1991). Changes in fibrinolytic factors in newborns during extracorporeal membrane oxygenation (ECMO). Am. J. Hematol..

[52] Ghiselli G. (2019). Heparin Binding Proteins as Therapeutic Target: An Historical Account and Current Trends. Medicine.

[53] Boneu B., Caranobe C., Sie P. (1990). Pharmacokinetics of heparin and low molecular weight heparin. Bailliere’s Clin. Haematol..

[54] Wiggins R.C., Bouma B.N., Cochrane C.G., Griffin J.H. (1977). Role of high-molecular-weight kininogen in surface-binding and activation of coagulation Factor XI and prekallikrein. Proc. Natl. Acad. Sci. USA.

[55] DeLoughery E.P., Olson S.R., Puy C., McCarty O.J.T., Shatzel J.J. (2019). The Safety and Efficacy of Novel Agents Targeting Factors XI and XII in Early Phase Human Trials. Semin. Thromb. Hemost..

[56] Wallisch M., Lorentz C.U., Lakshmanan H.H.S., Johnson J., Carris M.R., Puy C., Gailani D., Hinds M.T., McCarty O.J.T., Gruber A. (2020). Antibody inhibition of contact factor XII reduces platelet deposition in a model of extracorporeal membrane oxygenator perfusion in nonhuman primates. Res. Pract. Thromb. Haemost..

[57] Dobrovolskaia M.A., McNeil S.E. (2015). Safe anticoagulation when heart and lungs are “on vacation”. Ann. Transl. Med..

