# Supplementary material for: The Role of Excessive Anticoagulation and Missing Hyperinflammation in ECMO-Associated Bleeding
Source: J Clin Med. 2022 Apr 21;11(9):2314. doi: 10.3390/jcm11092314 (PMC9102211; doi:10.3390/jcm11092314)
Supplement: Supplementary file 1 [file jcm-11-02314-s001.zip › jcm-1608743-supplementary.pdf]

Journal of
Clinical Medicine

MDPI

Article

The Role of Excessive Anticoagulation and Missing Hyperinflammation in ECMO-Associated Bleeding

Sasa Rajsic [1,†], Robert Breitkopf [2], Ulvi Cenk Oezpeker [3,*], Zoran Bukumirić [4], Moritz Dobesberger [1] and Benedikt Treml [1,†]

Supplementary Material

1. Main analysis

Table S1. One year mortality-related outcomes in patients with ECMO support (n = 321)

Table S2. Variability of laboratory values during the ECMO support period (n = 321)

Table S3. Risk factors for bleeding during ECMO support: univariate analysis (n = 321)

Table S4. Risk factors for bleeding, model including SOFA score and procalcitonin: Cox multivariate analysis (n = 321)

2. Subgroup analysis: Comparison of venoarterial and venovenous ECMO configuration

Table S5. Extracorporeal membrane oxygenation: patient demographics and clinical characteristics, comparison of venoarterial and venovenous configuration (n = 321)

Table S6. ECMO related characteristics and complications, comparison of venoarterial and venovenous configuration (n = 321)

Table S7. Laboratory parameters within 24 h prior to bleeding event and blood products substitution in regard to ECMO configuration (n = 321)

Table S8. Risk factors for bleeding: venoarterial ECMO configuration—univariate analysis

Table S9. Risk factors for bleeding: venovenous ECMO configuration—univariate analysis

Table S10. Risk factors for bleeding in regard to EMCO configuration: Cox multivariate analysis

3. Subgroup analysis: Comparison of ECMO patients with and without surgical intervention

Table S11. ECMO related characteristics and complications, comparison of patients with and without surgical intervention (n = 321)

Table S12. Risk factors for bleeding in regard to surgical intervention presence—Cox multivariate analysis (based on the univariate analysis, data not shown; n = 321)

4. Subgroup analysis: Comparison of ECMO patients with major bleeding and minor or no bleeding event

Table S13. Major bleeding versus no or minor bleeding event—patient demographics and clinical characteristics (n = 321)

Table S14. Risk factors for bleeding—major bleeding versus no or minor bleeding event; Cox multivariate analysis (based on the univariate analysis, data not shown; n = 321)

1. Main analysis

Supplementary Table S1. One year mortality-related outcomes in patients with ECMO support (n = 321)

Mortality-related outcomes	All patients (n = 321)	No bleeding event (n = 198)	Bleeding event (n = 123)	p-value	Missing data (n/total)
Time from ECMO initiation to death (within 90 days)	10.0 (1-87)	9.5 (1-79)	10.5 (1-87)	0.623	0/321
Death on ECMO	69 (21.5)	36 (18.2)	33 (26.8)	0.071	0/321
ICU—mortality	115 (35.8)	58 (29.3)	57 (46.3)	0.003	0/321
In-hospital mortality	124 (38.6)	65 (32.8)	59 (48.0)	0.009	0/321
1 month mortality	107 (33.3)	55 (27.8)	52 (42.3)	0.010	0/321
2 months mortality	124 (38.6)	63 (31.8)	61 (49.6)	0.002	0/321
3 months mortality	126 (39.3)	64 (32.3)	62 (50.4)	0.002	0/321
6 months mortality	130 (40.5)	68 (34.3)	62 (50.4)	0.005	0/321
One year mortality	132 (41.1)	69 (34.8)	63 (51.2)	0.005	0/321
Cause of death (In-hospital mortality, n = 124)					0/124
Multiple organ failure	38 (30.7)	18 (27.7)	20 (33.9)		
Cardiac cause	41 (33.1)	20 (30.8)	21 (35.6)		
Respiratory failure	6 (4.8)	3 (4.6)	3 (5.1)	0.724	
Sepsis	13 (10.5)	8 (12.3)	5 (8.5)		
Brain death	26 (21.0)	16 (24.6)	10 (16.9)		

*Data presented as median (minimum – maximum range) or number of patients (%). Abbreviations: ICU: intensive care unit; ECMO: extracorporeal membrane oxygenation.

Analysis of laboratory parameter fluctuations

The analysis of laboratory parameter fluctuations during the whole period of ECMO support demonstrated a higher variability of prothrombin time (HR=1.02, p=0.016) being associated with bleeding (Supplementary table 1.2.). To the best of our knowledge, this was the first study reporting on variability analysis during ECMO support. This higher variability could be a result of more deteriorated coagulation status and its consequent correction trough substitution. The analysis of variability only before bleeding events in our cohort was not possible, as the median day of bleeding was the second ECMO day, giving not enough space for calculation over the period. Further studies with a larger patient cohort should explore if variability of laboratory parameters before events of interest can be utilised as predictors.

Supplementary Table S2. Variability of laboratory values during the ECMO support period (n = 321)

Laboratory parameter	B-coefficient	P-value	HR	95% CI lower	95% CI upper
C-reactive protein (mg/L)	0.005	0.157	1.01	0.99	1.01
Procalcitonin (µg/L)	0.001	0.681	1.00	0.99	1.01
White blood cells (g/L)	0.002	0.758	1.00	0.99	1.02
Platelets (g/L)	0.001	0.755	1.00	0.99	1.01
Prothrombin time (%)	0.015	0.016	1.02	1.00	1.03
International normalised ratio	0.009	0.069	1.01	0.99	1.02

Activated partial thromboplastin time (s)	0.004	0.290	1.00	1.99	1.01
Fibrinogen (mg/dL)	0.004	0.571	1.00	0.99	1.02
Antithrombin (%)	0.004	0.581	1.00	0.99	1.02
Factor XIII (%)	0.007	0.350	1.01	0.99	1.02
InTEM clotting time (s)	0.012	0.189	1.00	0.99	1.03
InTEM maximal clot firmness (mm)	0.009	0.560	1.01	0.98	1.04
FibTEM maximal clot firmness (mm)	-0.001	0.867	0.99	0.95	1.02
InTEM lysis index (%)	-0.007	0.775	0.99	0.95	1.04
ExTEM clotting time (mm)	0.005	0.546	1.05	0.99	1.12

*Event presents bleeding, or the laboratory value on a second day for patients without bleeding. Abbreviations: CI: confidence intervals; HR: hazard ratio; ECMO: extracorporeal membrane oxygenation

Supplementary Table S3. Risk factors for bleeding during ECMO support: univariate analysis (n = 321)

Nondependent variable	B-coefficient	P-value	HR	95% CI		Missing data (n/total)
				lower	upper	
Age (years)	-0.002	0.750	0.998	0.99	1.01	0/321
Sex (male/female)	0.054	0.786	1.06	0.71	1.56	0/321
Height (cm)	-1.010	0.214	0.36	0.07	1.80	10/321
Weight (kg)	-0.005	0.378	0.99	0.99	1.01	10/321
Body mass index (kg/m^2)	-0.007	0.691	0.99	0.96	1.03	10/321
SAPS III score	0.013	0.030	1.01	1.00	1.02	1/321
SOFA score	0.063	0.007	1.07	1.02	1.12	0/321
Reanimation before ECMO	0.289	0.193	1.34	0.87	2.06	0/321
ICU Department	0.103	0.568	1.11	0.78	1.58	0/321
Weekend	-0.264	0.272	0.77	0.48	1.23	0/321
ICU length of stay	-0.001	0.887	1.00	0.99	1.01	0/321
ECMO duration	-0.020	0.300	0.98	0.94	1.02	0/321
Admission reason (reference category: respiratory disease)						0/321
Cardiac non-surgical	0.258	0.252	1.30	0.83	2.01	
Cardiac surgery	0.305	0.274	1.36	0.79	2.34	
Trauma	0.602	0.555	1.83	0.25	13.48	
Hypothermia	0.486	0.366	1.63	0.57	4.67	
Type of ECMO	-0.268	0.158	0.77	0.53	1.11	
ECMO Indication (reference category: respiratory failure)						0/321
Cardiogenic shock	0.261	0.213	1.30	0.86	1.96	

Re-warming	0.590	0.270	1.81	0.63	5.15	
Complications						
Thrombosis	-0.118	0.587	0.89	0.58	1.36	0/321
Coagulopathy	0.318	0.178	1.38	0.87	2.19	27/321
Haemorrhage						0/321
Sepsis	0.153	0.462	1.17	0.78	1.75	0/321
Laboratory values 24 h prior to event*						
C-reactive protein (mg/L)	-0.036	0.003	0.96	0.94	0.99	15/321
Procalcitonin (µg/L)	-0.010	0.047	0.99	0.99	1.00	30/321
White blood cells (g/L)	-0.009	0.526	0.99	0.96	1.01	13/321
Platelets (g/L)	0.000	0.876	1.00	0.99	1.00	13/321
Prothrombin time (%)	-0.006	0.225	0.99	0.99	1.00	10/321
International normalised ratio	0.187	0.064	1.21	0.99	1.47	11/321
Activated partial thromboplastin time (s)	0.007	0.001	1.01	1.00	1.01	21/321
Fibrinogen (mg/dL)	-0.001	0.092	0.99	0.99	1.00	15/321
Antithrombin (%)	0.008	0.107	1.01	0.99	1.02	14/321
Factor XIII (%)	0.008	0.163	1.01	0.99	1.02	188/321
InTEM clotting time (s)	0.003	0.007	1.00	1.00	1.01	191/321
InTEM maximal clot firmness (mm)	-0.014	0.336	0.99	0.96	1.01	216/321
FibTEM maximal clot firmness (mm)	-0.036	0.146	0.96	0.92	1.01	217/321
InTEM lysis index (%)	-0.011	0.498	0.99	0.96	1.02	216/321
ExTEM clotting time (mm)	0.002	0.059	1.00	1.00	1.00	216/321

*Event presents bleeding, or the laboratory value on a second day for patients without bleeding. Abbreviations: CI: confidence intervals; HR: hazard ratio; SAPS III: simplified acute physiology score III; SOFA: sequential organ failure assessment score; ICU: intensive care unit; and ECMO: extracorporeal membrane oxygenation

Supplementary Table S4. Risk factors for bleeding, model including SOFA score and procalcitonin: Cox multivariate analysis (n = 321)

Nondependent variable	B-coefficient	P-value	HR	95% confidence interval	
				lower	upper
SOFA score	0.058	0.033	1.01	1.00	1.12
Procalcitonin (µg/L)	-0.012	0.029	0.99	0.98	1.00
aPTT (s)	0.006	0.035	1.01	1.00	1.01
Fibrinogen (mg/dL)	0.001	0.611	1.00	1.00	1.00

Abbreviations: SOFA: sequential organ failure assessment score; aPTT: activated partial thromboplastin time. (Cases with missing data: 40/321)

2. **Subgroup analysis: Comparison of venoarterial and venovenous ECMO configuration**

In the subgroup analysis comparing venoarterial and venovenous ECMO configurations, five patients were admitted to the ICU due to cardiac condition, developed respiratory failure, and needed VV-ECMO. Moreover, 14 patients with respiratory failure as the main ECMO indication received VA-ECMO due to the joint circulatory shock.

Supplementary Table S5. Extracorporeal membrane oxygenation: patient demographics and clinical characteristics, comparison of venoarterial and venovenous configuration (n = 321)

Patient characteristics	All patients (n = 321)	Venoarterial ECMO (n = 247)	Venovenous ECMO (n = 74)	p-value	Missing data (n/total)
Age (years)	57.5 ±16.1	60.4 ±15.0	47.7 ±16.1	<0.001	0/321
Male sex	229 (71.3)	170 (68.8)	59 (79.7)	0.069	0/321
Height (cm)	173 ±10.0	172 ±9.3	173 ±11.9	0.497	10/321
Weight (kg)	81.6 ±16.2	80.6 ±16.3	84.7 ±22.2	0.088	10/321
Body mass index (kg/m^2)	27.3 ±5.3	27.0 ±4.7	28.2 ±7.0	0.106	10/321
SAPS III score (points)	67 (28-117)	67 (28-104)	67 (31-117)	0.241	1/321
SAPS III score predicted mortality (%)	50 (1-96)	50 (1-93)	50 (2-96)	0.240	1/321
SOFA score (points)	12 (2-21)	12 (3-20)	13 (2-21)	0.521	0/321
SOFA respiratory	2 (0-4)	2 (0-4)	4 (0-4)	<0.001	
SOFA coagulation	1 (0-4), mean 1.2	1 (0-4), mean 1.3	1 (0-4), mean 0.9	0.006	
SOFA liver	0 (0-4)	1 (0-4)	0 (0-4)	0.246	
SOFA cardiovascular	4 (0-4)	4 (0-4)	3 (0-4)	<0.001	
SOFA neurology	4 (0-4)	4 (0-4)	4 (0-4)	0.204	
SOFA renal	1 (0-4)	1 (0-4)	0.5 (0-4)	0.404	
CPR before ECMO initiation	61 (19.0)	54 (21.9)	7 (9.5)	0.017	0/321
Length of ICU stay (days)	18 (1-170)	17 (1-170)	20.5 (2-98)	0.010	0/321
ICU admission reason					0/321
Respiratory failure	79 (24.6)	14 (5.7)	65 (87.8)		
Cardiac non-surgical	166 (51.7)	161 (65.2)	5 (6.8)		
Cardiac surgery	61 (19.0)	61 (24.7)	0 (0.0)	<0.001	
Trauma	3 (0.9)	1 (0.4)	2 (2.7)		
Hypothermia	12 (3.7)	10 (4.0)	2 (2.7)		
ICU department					0/321
ICU 1	178 (55.5)	145 (58.7)	33 (44.6)	0.032	
ICU 2	143 (44.5)	102 (41.3)	41 (55.4)		
Mortality-related outcomes					0/321
Time from admission to death within 90 days (days)	10 (1-88)	10 (1-79)	20 (2-88)	0.033	
ICU mortality	115 (35.8)	91 (36.8)	24 (32.4)	0.488	

*Data presented as mean ± standard deviation, median (minimum – maximum range) or number of patients (%). For clarity, mean is added if median is 0 and p value <0.05. Abbreviations: SAPS III: simplified acute physiology score III; SOFA: sequential organ failure assessment score; ICU: intensive care unit; ECMO: extracorporeal membrane oxygenation; CPR: cardiopulmonary resuscitation; ICU 1: general and surgical ICU; and ICU 2: traumatology ICU

Supplementary Table S6. ECMO-related characteristics and complications, comparison of venoarterial and venovenous configuration (n = 321)

Clinical characteristics	All patients (n = 321)	Venoarterial ECMO (n = 247)	Venovenous ECMO (n = 74)	p-value	Missing data (n/total)
ECMO support indications					0/321
Cardiogenic shock	223 (69.5)	223 (90.3)	0 (0.0)		
Respiratory failure	87 (27.1)	14 (5.7)	73 (98.6)	<0.001	
Re-warming	11 (3.4)	10 (4.0)	1 (1.4)		
ECMO related clinical course					
ECMO support duration (days)	6 (1-36)	6 (1-17)	8 (1-36)	<0.001	0/321
ECMO support duration <7 days	209 (65.1)	179 (72.5)	30 (40.5)	<0.001	0/321
Time from admission to ECMO initiation (days)	0 (0-36), mean 0.6	0 (0-20), mean 0.3	0 (0-36), mean 1.5	0.004	0/321
Day of ECMO support initiation					0/321
Weekday	255 (79.4)	193 (78.1)	62 (83.8)	0.239	
Weekend	66 (20.6)	54 (21.9)	12 (16.2)		
Anticoagulation during ECMO support					1/321
None	29 (9.1)	24 (9.7)	5 (6.8)		
UFH	256 (80.0)	197 (79.8)	59 (80.8)		
Argatroban	30 (9.4)	23 (9.3)	7 (9.6)	0.476	
Epoprostenol	1 (0.3)	0 (0)	1 (1.4)		
Argatroban and Epoprostenol	4 (1.3)	3 (1.2)	1 (1.4)		
Complications					
Major haemorrhage	60 (18.7)	41 (16.6)	19 (25.7)	0.079	0/321
Minor haemorrhage	63 (19.6)	52 (21.1)	11 (14.9)	0.240	0/321
Day of haemorrhage	5 (1-14)	4 (1-14)	6 (1-14)	0.001	0/321
Haemorrhage on the first ECMO day	52 (16.2)	46 (18.6)	6 (8.1)	0.031	0/321
Coagulopathy	41 (12.8)	28 (11.3)	13 (17.6)	0.159	27/321
Thrombosis	74 (23.1)	58 (23.5)	16 (21.6)	0.739	0/321
Sepsis	67 (20.9)	34 (13.8)	33 (44.6)	<0.001	0/321
Reason for ECMO support termination					0/321
Improvement (weaned)	230 (71.7)	174 (70.4)	56 (75.7)		
Bridge to other assistance (heart transplant or ventricular assist device)	17 (6.9)	17 (6.9)	0 (0.0)	0.074	
Haemorrhage	7 (2.2)	6 (2.4)	1 (1.4)		
Death	67 (20.9)	50 (20.2)	17 (23.0)		

*Data presented as median (minimum – maximum range) or number of patients (%). For clarity, mean is added if median is 0 and p value <0.05. Abbreviations: ECMO: extracorporeal membrane oxygenation, UFH: unfractionated heparin.

Supplementary Table S7. Laboratory parameters within 24 h prior to bleeding event and blood products substitution in regard to ECMO configuration (n = 321)

Laboratory parameter	All patients (n = 321)	Venoarterial ECMO (n = 247)	Venovenous ECMO (n = 74)	p-value	Missing data (n/total)
Haemoglobin (g/dL)	92.6 ±13.3	92.4 ±12.5	93.0 ±15.6	0.778	13/321
Red blood cells (T/L)	3.2 ±0.5	3.1 ±0.5	3.2 ±0.5	0.138	13/321
Haematocrit (%)	0.3 ±0.1	0.3 ±0.1	0.3 ±0.1	0.121	13/321
Leucocytes (g/L)	10.2 (1.3-71.7)	9.8 (1.3-71.7)	11.5 (1.5-30.6)	0.022	13/321
C-reactive protein (mg/L)	7.0 (0.1-35.5)	6.1 (0.1-35.5)	15.7 (0.1-35.5)	<0.001	15/321
Procalcitonin (μg/L)	5.0 (0.1-1272.4)	5.0 (0.1-175.6)	4.6 (0.1-1272.4)	0.800	30/321
Platelets (g/L)	87.0 (14-309)	84.5 (14-309)	100.0 (18-292)	0.042	13/321
International normalised ratio	1.5 (0.8-6.0)	1.5 (0.8-6.0)	1.3 (1.0-6.0)	<0.001	11/321
Activated partial thromboplastin time (s)	37.0 (24-201)	36.0 (24-201)	42.0 (24-201)	0.312	21/321
Prothrombin time (%)	51.0 (9-104)	48.0 (9-101)	62.0 (9-104)	<0.001	10/321
Fibrinogen (mg/dL)	256.5 (39-1053)	250.0 (39-941)	396.0 (76-1053)	<0.001	15/321
Antithrombin (%)	45.0 (19-118)	44.0 (19-99)	51.0 (19-118)	0.008	14/321
Factor XIII (%)	59.0 (13-131)	61.0 (22-131)	45.5 (13-79)	0.001	188/321
InTEM clotting time (s)	211 (54-671)	213.5 (54-671)	197 (158-301)	0.202	191/321
InTEM maximal clot firmness (mm)	49 (5-71)	48 (5-71)	53.5 (35-64)	0.053	216/321
FibTEM maximal clot firmness (mm)	14 (2-31)	13 (2-31)	17 (4-30)	0.331	217/321
Substitution of blood products during ECMO support					
Packed red blood cells (units)	5.0 (0-60)	6.0 (0-60)	3.5 (0-33)	0.155	15/321
Fresh-frozen plasma (units)	0 (0-92), mean 2.7	0 (0-92), mean 3.4	0 (0-10), mean 0.5	<0.001	15/321
Platelets (units)	1 (0-30)	1 (0-22)	0 (0-30)	0.028	15/321
Fibrinogen (g)	0 (0-26), mean 2.8	0 (0-26), mean 3.0	0 (0-26), mean 2.3	0.033	15/321
Antithrombin (IU)	0 (0-32000), mean 614.8	0 (0-17266), mean 501.5	0 (0-32000), mean 993.2	0.823	15/321
Prothrombin complex concentrate (IU)	0 (0-7200), mean 386.0	0 (0-7200), mean 440.1	0 (0-4500), mean 205.4	0.042	15/321
Factor XIII concentrate (IU)	0 (0-10000), mean 570.3	0 (0-6750), mean 492.9	0 (0-10000), mean 827.7	0.058	15/321
Desmopressin (μg)	0 (0-30), mean 0.6	0 (0-30), mean 0.4	0 (0-30), mean 0.6	0.206	18/321
Von Willebrand factor (IU)	0 (0-5000), mean 100.3	0 (0-5000), mean 80.9	0 (0-4000), mean 164.9	0.180	27/321

*Data presented as mean ± standard deviation, median (minimum – maximum range) or number of patients (%). For clarity, mean is added if median is 0 and p value <0.05. Abbreviations: ECMO: extracorporeal membrane oxygenation; IU: international units

Supplementary Table S8. Risk factors for bleeding: venoarterial ECMO configuration—univariate analysis

Nondependent variable	B-coefficient	P-value	HR	95% CI	
				lower	upper
Age (years)	-0.003	0.670	0.99	0.98	1.01
Sex (male/female)	0.010	0.965	0.99	0.65	1.54
Body mass index (kg/m^2)	-0.018	0.430	0.98	0.94	1.03
SAPS III score	0.014	0.039	1.01	1.00	1.03
SOFA score	0.066	0.023	1.07	1.01	1.13
Reanimation before ECMO	0.291	0.221	1.34	0.84	2.13
Weekend	-0.465	0.100	0.63	0.36	1.09
ICU length of stay	-0.001	0.887	1.00	0.99	1.01
ECMO duration	-0.020	0.300	0.98	0.94	1.02
Previous surgical procedure	-0.393	0.060	0.68	0.45	1.02
Complications					
Thrombosis	-0.177	0.484	0.84	0.51	1.38
Coagulopathy	0.235	0.432	1.27	0.70	2.27
Sepsis	-0.146	0.636	0.86	0.47	1.59
Laboratory values 24h prior to event*					
C-reactive protein (mg/L)	-0.056	0.003	0.95	0.91	0.98
Procalcitonin (μg/L)	-0.012	0.086	0.99	0.98	1.00
White blood cells (g/L)	-0.023	0.251	0.98	0.94	1.02
Platelets (g/L)	0.001	0.582	1.00	0.99	1.01
Prothrombin time (%)	-0.006	0.283	0.99	0.98	1.01
International normalised ratio	0.212	0.043	1.24	1.01	1.52
Activated partial thromboplastin time (s)	0.008	<0.001	1.01	1.00	1.01
Fibrinogen (mg/dL)	-0.001	0.238	0.99	0.99	1.00
Antithrombin (%)	0.008	0.207	1.01	0.99	1.02
Factor XIII (%)	0.007	0.277	1.01	0.99	1.02
InTEM clotting Time (s)	0.003	0.006	1.00	1.00	1.01
InTEM maximal clot firmness (mm)	-0.015	0.333	0.99	0.96	1.02
FibTEM maximal clot firmness (mm)	-0.045	0.138	0.96	0.90	1.01
InTEM lysis index (%)	-0.012	0.450	0.99	0.96	1.02
ExTEM clotting time (mm)	0.002	0.078	1.00	1.00	1.00

*Event presents bleeding, or the laboratory value on a second day for patients without bleeding. Abbreviations: CI—confidence intervals; HR—hazard ratio; SAPS III: simplified acute physiology score III; SOFA: sequential organ failure assessment score; ICU: intensive care unit; and ECMO: extracorporeal membrane oxygenation.

Supplementary Table S9. Risk factors for bleeding: venovenous ECMO configuration—univariate analysis

Nondependent variable	B-coefficient	P-value	HR	95% CI	
				lower	upper
Age (years)	-0.006	0.639	0.994	0.97	1.02
Sex (male/female)	0.235	0.591	1.26	0.54	2.98
Body mass index (kg/m^2)	0.014	0.614	1.01	0.96	1.07
SAPS III score	0.009	0.420	1.01	1.00	1.03
SOFA score	0.055	0.182	1.06	0.97	1.15
Reanimation before ECMO	0.225	0.763	1.25	0.29	5.38
Weekend	0.502	0.272	1.65	0.67	4.05
ICU length of stay	-0.001	0.887	1.00	0.99	1.01
ECMO duration	-0.020	0.300	0.98	0.94	1.02
Previous surgical procedure	0.119	0.783	1.13	0.48	2.64
Complications					
Thrombosis	0.123	0.777	1.13	0.48	2.65
Coagulopathy	0.461	0.258	1.59	0.71	2.53
Sepsis	0.834	0.028	2.30	1.09	4.84
Laboratory values 24 h prior to event*					
C-reactive protein (mg/L)	-0.019	0.290	0.98	0.95	1.02
Procalcitonin (µg/L)	-0.007	0.231	0.99	0.98	1.00
White blood cells (g/L)	0.020	0.406	1.02	0.97	1.07
Platelets (g/L)	-0.003	0.357	1.00	0.99	1.00
Prothrombin time (%)	-0.005	0.623	0.99	0.98	1.01
International normalised ratio	-0.186	0.688	0.83	0.335	2.06
Activated partial thromboplastin time (s)	0.003	0.698	1.00	1.00	1.02
Fibrinogen (mg/dL)	-0.002	0.218	0.99	0.99	1.00
Antithrombin (%)	0.010	0.227	1.01	0.99	1.03
Factor XIII (%)	0.018	0.257	1.02	0.99	1.05
InTEM clotting time (s)	-0.003	0.757	1.00	1.00	1.02
InTEM maximal clot firmness (mm)	-0.027	0.449	0.98	0.91	1.04
FibTEM maximal clot firmness (mm)	-0.049	0.332	0.95	0.86	1.05
InTEM lysis index (%)	-0.065	0.479	0.94	0.78	1.12
ExTEM clotting time (mm)	0.003	0.906	1.00	0.95	1.06

*Event presents bleeding, or the laboratory value on a second day for patients without bleeding. Abbreviations: CI: confidence intervals; HR: hazard ratio; SAPS III: simplified acute physiology score III; SOFA: sequential organ failure assessment score; ICU: intensive care unit; and ECMO: extracorporeal membrane oxygenation

Supplementary Table S10. Risk factors for bleeding in regard to EMCO configuration: Cox multivariate analysis

Nondependent variable	B-coefficient	P-value	HR	95% confidence interval	
				lower	upper
Venoarterial ECMO					
SAPS III score	0.013	0.081	1.01	0.99	1.03
Surgical intervention	-0.312	0.161	0.73	0.47	1.13
C-reactive protein (mg/L)	-0.042	0.020	0.96	0.93	0.99
aPTT (s)	0.005	0.029	1.01	1.00	1.01
Venovenous ECMO					
SAPS III score	0.010	0.385	1.01	0.99	1.03
Surgical intervention	0.268	0.555	1.31	0.54	3.19
C-reactive protein (mg/L)	-0.022	0.226	0.98	0.95	1.01
aPTT (s)	0.002	0.765	1.00	0.99	1.02

Abbreviations: ECMO: extracorporeal membrane oxygenation; SAPS III: simplified acute physiology score III; and aPTT: activated partial thromboplastin time. (Cases with missing data: 21/321)

3. Subgroup analysis: Comparison of ECMO patients with and without surgical intervention

In the group of patients with surgical intervention, we included all patients having surgery, other than ECMO cannulation (e.g. postcardiotomy, abdominal surgeries, polytrauma being surgically treated, etc.).

Supplementary Table S11. ECMO-related characteristics and complications, comparison of patients with and without surgical intervention (n = 321)

Clinical characteristics	All patients (n = 321)	No surgical intervention (n = 147)	Surgical intervention (n = 174)	p-value	Missing data (n/total)
Age (years)	57.5 ±16.1	51.8 ±15.6	62.3 ±14.9	<0.001	0/321
Male sex	229 (71.3)	113 (76.9)	116 (66.7)	0.044	0/321
SAPS III score (points)	67.0 (28-117)	69.5 (28-117)	64.0 (28-100)	<0.001	1/321
CPR before ECMO initiation	61 (19.0)	40 (27.2)	21 (12.1)	0.001	0/321
Length of ICU stay (days)	18 (1-170)	15 (1-170)	20 (2-121)	0.041	0/321
ECMO support indications					0/321
Cardiogenic shock	223 (69.5)	76 (51.7)	147 (84.5)	<0.001	
Respiratory failure	87 (27.1)	60 (40.8)	27 (15.5)		
Re-warming	11 (3.4)	11 (7.5)	0 (0.0)		
Type of ECMO support					0/321
Venoarterial	247 (76.9)	92 (62.6)	155 (89.1)	<0.001	
Venovenous	74 (23.1)	55 (37.4)	19 (10.9)		
ECMO related clinical course					
ECMO support duration (days)	6 (1-36)	7 (1-30)	6 (1-36)	0.003	0/321
ECMO support duration <7 days	209 (65.1)	87 (55.1)	128 (73.6)	0.001	0/321
Anticoagulation during ECMO support					1/321

None	29 (9.1)	13 (8.8)	16 (9.2)		
UFH	256 (80.0)	115 (78.2)	141 (81.5)		
Argatroban	30 (9.4)	16 (10.5)	14 (8.1)	0.805	
Epoprostenol	1 (0.3)	1 (0.7)	0 (0.0)		
Argatroban and Epoprostenol	4 (1.3)	2 (1.4)	2 (1.2)		
Complications					
Haemorrhage	123 (38.3)	65 (44.2)	58 (33.3)	0.046	0/321
Major haemorrhage	60 (18.7)	22 (15.0)	38 (21.8)	0.116	0/321
Minor haemorrhage	63 (19.6)	43 (29.3)	20 (11.5)	<0.001	0/321
Coagulopathy	41 (12.8)	24 (16.3)	17 (9.8)	0.080	27/321
Thrombosis	74 (23.1)	30 (20.4)	44 (25.3)	0.301	0/321
Sepsis	67 (20.9)	39 (26.5)	28 (16.1)	0.022	0/321
Laboratory values 24h prior to event*					
C-reactive protein (mg/L)	7.0 (0.1-35.5)	6.6 (0.1-35.5)	7.0 (0.1-34.8)	0.433	15/321
Procalcitonin (μg/L)	5.0 (0.1-1272)	3.7 (0.1-1272)	6.6 (0.1-1272)	0.009	30/321
Platelets (g/L)	87 (14-309)	112 (14-292)	76 (31-309)	<0.001	13/321
Activated partial thromboplastin time (s)	37 (24-201)	40 (24-201)	36 (24-201)	0.356	11/321

*Event presents bleeding, or the laboratory value on a second day for patients without bleeding. **Data presented as median (minimum – maximum range) or number of patients (%). For clarity, mean was added if median was 0 and p value <0.05. Abbreviations: ECMO: extracorporeal membrane oxygenation. UFH: unfractionated heparin.

Supplementary Table S12. Risk factors for bleeding in regard to surgical intervention presence—Cox multivariate analysis (based on the univariate analysis, data not shown; n = 321)

Nondependent variable	B-coefficient	P-value	HR	95% confidence interval	
				lower	upper
No surgical intervention					
SAPS III score	0.011	0.230	1.01	0.99	1.03
Reanimation before ECMO	0.281	0.368	1.32	0.72	2.44
C-reactive protein (mg/L)	-0.043	0.034	0.96	0.92	0.99
aPTT (s)	0.009	0.004	1.01	1.00	1.02
Fibrinogen (mg/dL)	0.001	0.379	1.00	0.99	1.00
Surgical intervention present					
SAPS III score	0.013	0.197	1.01	0.99	1.03
Reanimation before ECMO	-0.511	0.289	0.60	0.23	1.54
C-reactive protein (mg/L)	-0.056	0.054	0.95	0.89	1.00
aPTT (s)	-0.006	0.306	0.99	0.98	1.01
Fibrinogen (mg/dL)	0.002	0.150	1.00	0.99	1.01

Abbreviations: ECMO: extracorporeal membrane oxygenation; SAPS III: simplified acute physiology score III; and aPTT: activated partial thromboplastin time. (Cases with missing data: 21/321)

4. Subgroup analysis: Comparison of ECMO patients with minor or no bleeding and major bleeding event

We reported on bleeding events as recommended by ELSO guidelines. Even if different forms of haemorrhage were included (cannula insertion site, operative site, intracranial, etc.), it was possible to divide minor and major haemorrhage based on ELSO guidelines. Moreover, we performed subgroup analysis comparing all patients without or with minor bleeding events to those with major bleeding event.

Supplementary Table S13. Major bleeding versus no or minor bleeding event—patient demographics and clinical characteristics (n = 321)

Characteristics	All patients (n = 321)	Minor or no bleeding (n = 261)	Major bleeding (n = 60)	p-value	Missing data (n/total)
SAPS III score (points)	67 (28-117)	66 (28-117)	69 (31-104)	0.258	1/321
SAPS III score predicted mortality (%)	50 (1-96)	48 (1-96)	54 (2-93)	0.257	1/321
SOFA score (points)	12 (2-21)	12 (2-21)	13 (4-21)	0.004	0/321
Length of ICU stay (days)	18 (1-170)	17 (1-170)	19.5 (1-92)	0.131	0/321
ICU admission reason					0/321
Respiratory failure	79 (24.6)	60 (23.0)	19 (31.7)		
Cardiac non-surgical	166 (51.7)	142 (54.4)	24 (40.0)		
Cardiac surgery	61 (19.0)	45 (17.2)	16 (26.7)	0.137	0/321
Trauma	3 (0.9)	3 (1.1)	0 (0.0)		
Hypothermia	12 (4.0)	11 (4.2)	1 (1.7)		
ICU mortality	115 (35.8)	83 (31.8)	32 (53.3)	0.002	0/321
ECMO support indications					0/321
Cardiogenic shock	223 (69.5)	185 (70.9)	38 (63.3)		
Respiratory failure	87 (27.1)	66 (25.3)	21 (35.0)	0.251	
Re-warming	11 (3.4)	10 (3.8)	1 (1.7)		
Type of ECMO support					0/321
Venoarterial	247 (76.9)	206 (78.9)	41 (68.3)	0.079	
Venovenous	74 (23.1)	55 (21.1)	19 (31.7)		
ECMO related clinical course					0/321
ECMO support duration (days)	6 (1-36)	6 (1-30)	8 (1-36)	0.001	
ECMO duration <7 days	209 (65.1)	181 (69.3)	28 (46.7)	0.001	
Anticoagulation during ECMO support					1/321
None	29 (9.1)	22 (8.5)	7 (11.7)		
UFH	256 (80.0)	210 (80.8)	46 (76.7)		
Argatroban	30 (9.4)	25 (9.6)	5 (8.3)	0.291	
Epoprostenol	1 (0.3)	0 (0.0)	1 (1.7)		
Argatroban and Epoprostenol	4 (1.3)	3 (1.2)	1 (1.7)		
Complications					
Coagulopathy	41 (12.8)	27 (10.3)	14 (23.3)	0.007	27/321
Thrombosis	74 (23.1)	55 (21.1)	19 (31.7)	0.079	0/321
Sepsis	67 (20.9)	47 (18.0)	20 (33.3)	0.008	0/321

Reason for ECMO support termination					0/321
Improvement (weaned)	230 (71.7)	192 (73.6)	38 (63.3)	0.015	
Bridge to other assistance (heart transplant or ventricular assist device)	17 (5.3)	16 (6.1)	1 (1.7)		
Haemorrhage	7 (2.2)	3 (1.1)	4 (6.7)		
Death	67 (20.9)	50 (19.2)	17 (28.3)		

*Data presented as median (minimum – maximum range) or number of patients (%). For clarity, mean was added if median was 0 and p value <0.05. Abbreviations: SAPS III: simplified acute physiology score III; SOFA: sequential organ failure assessment score; ICU: intensive care unit; ECMO: extracorporeal membrane oxygenation; and UFH: unfractionated heparin.

Supplementary Table S14. Risk factors for bleeding—major bleeding versus no or minor bleeding event; Cox multivariate analysis (based on the univariate analysis, data not shown; n = 321)

Nondependent variable	B-coefficient	P-value	HR	95% confidence interval	
				lower	upper
No surgical intervention					
SOFA score	0.078	0.021	1.08	1.01	1.15
Surgical intervention	0.634	0.026	1.88	1.08	3.29
Sepsis	0.411	0.169	1.51	0.84	2.71
Coagulopathy	0.480	0.143	0.99	0.85	3.07

Abbreviations: ECMO: extracorporeal membrane oxygenation; SOFA: sequential organ failure assessment. (Cases with missing data: 21/321)
